# Bioreactor Parameters for Microcarrier-Based Human MSC Expansion under Xeno-Free Conditions in a Vertical-Wheel System

**DOI:** 10.3390/bioengineering7030073

**Published:** 2020-07-08

**Authors:** Josephine Lembong, Robert Kirian, Joseph D. Takacs, Timothy R. Olsen, Lye Theng Lock, Jon A. Rowley, Tabassum Ahsan

**Affiliations:** RoosterBio, Inc., 5295 Westview Drive, Suite 275, Frederick, MD 21703, USA; josephine@roosterbio.com (J.L.); robert@roosterbio.com (R.K.); joseph@roosterbio.com (J.D.T.); tim@roosterbio.com (T.R.O.); lyetheng@gmail.com (L.T.L.); jon@roosterbio.com (J.A.R.)

**Keywords:** bioreactor, hMSCs, microcarrier, bioprocess

## Abstract

Human mesenchymal stem/stromal cells (hMSCs) have been investigated and proven to be a well-tolerated, safe therapy for a variety of indications, as shown by over 900 registered hMSC-based clinical trials. To meet the commercial demand for clinical manufacturing of hMSCs, production requires a scale that can achieve a lot size of ~100B cells, which requires innovative manufacturing technologies such as 3D bioreactors. A robust suspension bioreactor process that can be scaled-up to the relevant scale is therefore crucial. In this study, we developed a fed-batch, microcarrier-based bioreactor process, which enhances media productivity and drives a cost-effective and less labor-intensive hMSC expansion process. We determined parameter settings for various stages of the culture: inoculation, bioreactor culture, and harvest. Addition of a bioreactor feed, using a fed-batch approach, was necessary to replenish the mitogenic factors that were depleted from the media within the first 3 days of culture. Our study resulted in an optimized hMSC culture protocol that consistently achieved hMSC densities between 2 × 10^5^–6 × 10^5^ cells/mL within 5 days with no media exchange, maintaining the final cell population doubling level (PDL) at 16–20. Using multiple hMSC donors, we showed that this process was robust and yielded hMSCs that maintained expansion, phenotypic characteristic, and functional properties. The developed process in a vertical-wheel suspension bioreactor can be scaled to the levels needed to meet commercial demand of hMSCs.

## 1. Introduction

Human mesenchymal stem/stromal cells (hMSCs) have been investigated and proven to be a well-tolerated, safe therapy for a variety of diseases [1], as indicated by over 900 registered hMSC-based clinical trials in ClinicalTrials.gov as of 2019. Previous analysis of cell dose estimates from the ClinicalTrials.gov website shows that most indications requiring hMSC treatment need a target production lot size of ~100B cells [1,2], with some exceptions including low dose indications such as ocular diseases [3]. Such production lot size requires a scalable manufacturing platform such as 3D bioreactors, where MSC expansion in suspension is typically performed by growing cells on adherent substrates such as microcarriers [4,5,6]. A robust suspension bioreactor process that can be scaled-up is therefore crucial to meet this demand for clinical manufacturing of hMSCs, and this starts with process development and optimization at a scaled-down bioreactor model.

To accommodate hMSC expansion in suspension bioreactors, it is critical to choose a bioreactor platform that allows cell attachment to the microcarriers, homogeneously suspends the cells and microcarriers, as well as distributes gas and nutrients while imposing low shear, as hMSCs are known to be sensitive to shear stress [7,8,9]. In addition to providing large cell numbers, an hMSC manufacturing platform should facilitate maintenance of the hMSC undifferentiated cell state and phenotype to retain their therapeutic potency. Among many bioreactor platforms, the stirred-tank bioreactor has been extensively evaluated for hMSC expansion [10,11,12,13]. More recently, vertical-wheel bioreactors have been developed to provide a uniform, low-shear fluid mixing environment for efficient particle suspension. The agitation mechanics provided by the vertical impeller and the shape of the bioreactor vessel allows for a better homogenization of the microcarriers with low power input compared to commonly used cylindrical bioreactors with horizontal impellers [14]. The low power, and consequently low hydrodynamic shear, makes this bioreactor specifically a great candidate for culture of shear-sensitive, anchorage dependent cells such as hMSCs [15,16]. In addition, this low shear environment remains constant across the full range of vessel sizes from 0.5 to 500 L [17]. For hMSC culture, it has been reported that the vertical-wheel bioreactor facilitated higher cell-microcarrier attachment compared to the stirred-tank bioreactor and the generated hMSCs showed a higher percentage of proliferative cells, lower percentage of apoptotic cells, and reduced levels of HLA-DR positive cells compared to cells grown on stirred-tank bioreactors [18]. The availability of this bioreactor platform in multiple scales, currently ranging from 0.1 L to 80 L, is also key as hMSC manufacturing processes need to be developed with future scalability in mind. Thus, a low shear, vertical-wheel bioreactor was chosen as the platform to develop our process. 

In clinical hMSC manufacturing, there has been movement away from serum-containing processes due to higher risk of graft rejection, immunoreactions, and virus contamination associated with animal-derived products [19,20,21]. To ensure product safety, xeno-free (XF) supplements such as human serum and human platelet lysate [19,22,23,24] have therefore become increasingly incorporated in hMSC clinical manufacturing processes [21]. While XF MSC-microcarrier cultures have been developed, it typically involves full or partial media exchanges to manage nutrient supply and waste build-up [25,26,27,28,29]. Borrowing from best practices in the monoclonal antibody field, fed batch processes can lead to high media productivity [30], as defined in the Glossary for Cell & Gene Therapy and Regenerative Medicine as millions of cells/liter of media consumed [31]. Optimizing this media productivity is critical as media is the major cost driver in cell manufacturing [30], which also carries the hidden costs of labor associated with media exchange. 

Another paradigm for consideration is perfusion, in which media is continuously circulated through the culture, therefore simultaneously supplying nutrients and removing waste from the culture. While perfusion offers a continuous process and can achieve high cell densities, it utilizes large quantities of media and consequently induces high media costs and logistics/supply chain issues [32]. Thus, perfusion has not been widely adopted at larger scales. In addition, most hMSC culture process development and pilot scale manufacturing utilize a fed-batch paradigm [33], making it a bigger regulatory hurdle to switch to a perfusion process and demonstrate comparability [32]. For this reason, a fed-batch process was developed in this study with future scalability in mind. 

**Figure 1:**
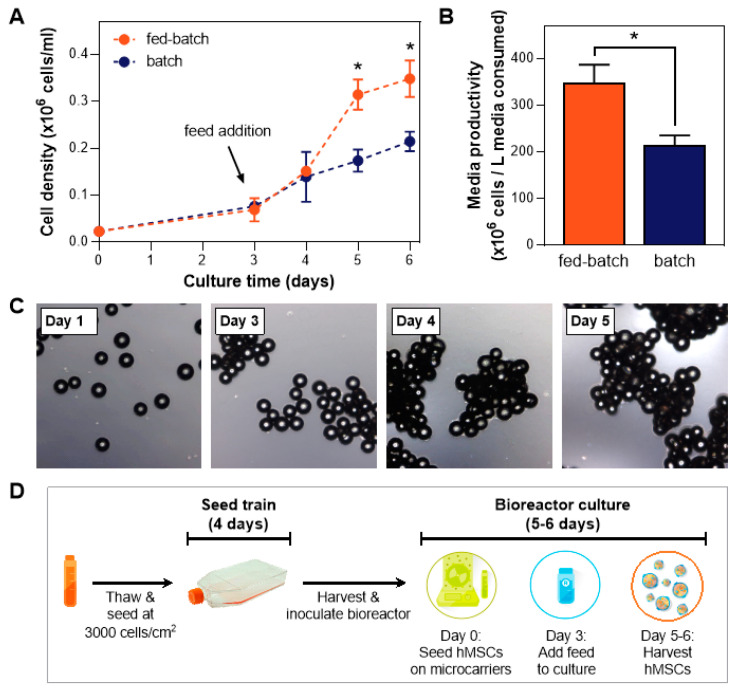
Fed-batch bioreactor culture paradigm. (**A**) Comparison study of a xeno-free (XF) bioreactor process utilizing batch and fed-batch processes shows a distinct advantage of the fed-batch process on final cell yield (*n* = 3). (**B**) Media productivity is enhanced using a fed-batch process. (**C**) Images indicating typical cell growth on microcarriers during a 5-day culture in a vertical-wheel bioreactor (* *p* ≤ 0.05). (**D**) Schematic of the bioreactor culture workflow using a fed-batch process.

We established this fed-batch process in hMSC expansion in the vertical-wheel bioreactors, where a fed-batch process showed a higher cell yield and therefore enhanced media productivity compared to a batch process (Figure 1A,B). Addition of the bioreactor feed was necessary to replenish the growth factors that were depleted from the media within the first 3 days of culture (Figure 1A). Formation of cell-bead aggregates during the expansion process was observed (Figure 1C), with aggregate size increasing over time.

With the fed-batch paradigm in place, further optimization of this MSC bioreactor culture process is central to maximizing yields and recovering healthy, functional cells at harvest. To achieve that, optimization studies need to be performed in the small-scale bioreactors prior to scaling up to a full production scale. Various stages of a XF bioreactor culture need to be studied: cell and microcarrier inoculation, seeding strategies, culture, feed, and harvest protocol (Figure 1D). Each of these steps is associated with numerous parameters that need to be evaluated, which is the objective of our study. In this paper, we developed and optimized a xeno-free, fed-batch, microcarrier-based culture process in a scalable low-shear bioreactor system, resulting in consistent hMSC yield and maintenance of cell health and characteristics following culture.

## 2. Materials and Methods 

### 2.1. hMSC Planar Culture for Seed Train

hBM-MSC Working Cell Banks (WCB) from five adult donors (RoosterBio, Frederick, MD, USA) were used in this study. These cells had been isolated and previously expanded under XF conditions (PDL: 8-10, Passage number: 2). Cells were thawed and seeded at a density of 3000 cells/cm^2^ in Corning^®^ CellBIND^®^ culture flasks (Corning Incorporated, Corning, NY, USA), in the accompanying High Performance XF Media Kit (RoosterBio). Cells were cultured at 37 °C with 5% CO_2_. After 4 days, cells were dissociated from the flasks with TrypLE™ Select Enzyme (Thermo Fisher Scientific, Waltham, MA, USA), quenched with medium, then counted using the NucleoCounter^®^ NC-200™ (Chemometec, Allerod, Denmark). Cell suspension was then spun down at 300× *g* for 5 min and resuspended in growth medium at a density of 1 × 10^6^ cells/mL for cell inoculation in the following steps.

### 2.2. hMSC Bioreactor Culture

Following the seed train, hMSCs were cultured on Corning^®^ Low Concentration Synthemax™ II microcarriers (Corning) inside PBS-0.1 single-use Vertical-Wheel™ bioreactors (PBS Biotech, Camarillo, CA, USA). To seed the cells onto the microcarriers, a 1–6.3 mL cell suspension was inoculated into the bioreactor containing 30 mL of medium and 0.75–2.5 g of microcarriers, resulting in cell seeding density of 11,000–70,000 cells/mL, and areal density of 2222–9333 cells/cm^2^ of microcarrier surface. To facilitate attachment, the cells were incubated for 20 min at static condition, then gently shaken to re-distribute the cells and the microcarriers, and finally incubated for 20 more minutes to further cell attachment to microcarriers. Our data showed that an average of 84% cell attachment could be achieved using this seeding method (Figure S1).

At the end of the cell attachment duration, fresh culture medium was added to a total volume of 90 mL. The PBS-0.1 vessel was placed on its controller base in a 5% CO_2_ incubator and agitation was initiated at 25 rpm. On Day 3 of the bioreactor culture, an XF bioreactor feed, RoosterReplenish™-MSC-XF (RoosterBio), was added at 2% of the working volume and agitation speed was increased to 30 rpm.

### 2.3. Bioreactor Sampling

Daily sampling of the bioreactor was performed to quantify cell numbers as well as the metabolite and waste profile to assess the kinetics of cell growth and media consumption. A 3 mL sample was taken daily while the cells/microcarriers were in suspension at 30 rpm agitation. The cell/microcarrier sample were left to sediment, the supernatant was isolated for medium analysis, and an equal volume of TrypLE was added to dissociate the cells from the microcarriers. Following dissociation, the number of cells was counted. The medium supernatant was analyzed using the BioProfile FLEX2 Analyzer (Nova Biomedical, Waltham, MA, USA) to monitor the metabolite and waste concentration.

Visualization of cell attachment and cell/microcarrier aggregation was performed by obtaining a 1 mL sample from the bioreactor and visualizing it under light microscopy (EVOS M5000 Cell Imaging System; ThermoFisher Scientific).

### 2.4. In-Vessel Harvest

Following hMSC expansion in bioreactors for 5–6 days, in-vessel harvest was performed. While bioreactor sampling provides the kinetics during culture, the most accurate quantification of total cell yield after the culture duration is obtained through complete in-vessel harvest, minimizing any potential errors from non-representative sampling when large cell-microcarrier aggregates are present in the culture. After agitation is stopped and the cells/microcarriers settle to the bottom of the bioreactor, the medium was removed and the remaining cells/microcarrier solution were washed with PBS. TrypLE was added to the bioreactor at half of the working volume, and agitation was initiated (20–100 rpm for 20–40 min) in a 37 °C incubator. Cells were then separated from the microcarriers using a 100-μm cell strainer, quenched with an equal volume of medium, and then counted to obtain the final harvest yield. Values are presented as concentrations (cells/mL) to emphasize the scalable process that was being developed.

### 2.5. Analysis of Cell Health, Quality Attributes and Functionality

Evaluation of hMSC properties was conducted on the cells harvested from the bioreactor and compared to the properties of cells that were expanded on 2D planar flasks, which served as a control. Cell populations were evaluated for expression of MSC surface markers and differentiation potential (to the adipo-, osteo-, and chondrogenic lineages). Functional properties of MSCs were also evaluated, such as immunomodulatory function and angiogenic cytokine secretion. 

#### 2.5.1. Cell Health Analysis

To determine the health of the hMSCs harvested from the bioreactor, cell expansion was quantified by culturing the cells harvested from the bioreactor in CellBIND flasks for 5 days. Following a 5-day culture, cells were harvested and the fold expansion was quantified. Expansion capability of the cells harvested from the bioreactor is an important measure of cell quality/health and is an important validation of the bioreactor process following the final cell yield.

#### 2.5.2. Tri-Lineage Differentiation

Qualitative evaluation of hMSC differentiation down adipo-, osteo-, and chondrogenic lineages were performed on the cells harvested from the bioreactor and compared to those from the 2D flask controls. For adipogenic differentiation, harvested cells were cultured in STEMPRO^®^ Adipogenesis Differentiation Kit media (Gibco) for 7–14 days, then fixed and stained with Oil Red O Solution (Sigma-Aldrich). For osteogenic differentiation, cells were cultured in STEMPRO^®^ Osteogenesis Differentiation Kit media (Gibco) for 14 days, then fixed and stained with 2% Alizarin Red Stain (Lifeline Cell Technology, Walkersville, MD, USA). For chondrogenic differentiation, cell pellets were generated in ultra-low attachment plates and cultured in STEMPRO^®^ Chondrogenesis Differentiation Kit media (Gibco) for 21 days, then fixed, sectioned, and stained with Alcian Blue. All differentiated samples were imaged following staining and compared with matched undifferentiated samples. 

#### 2.5.3. Flow Cytometry for Cell Surface Marker Expression

Cells dissociated from the microcarriers and harvested from the bioreactors were analyzed for the expression of surface markers (CD73(+), CD90(+), CD105(+), CD166(+), CD14(−), CD34(−), CD45(−)) using flow cytometry and compared to those from 2D flask controls. 

#### 2.5.4. Immunomodulatory Function

Induction of indoleamine 2,3-dioxygenase (IDO) activity by exposure of hMSCs to the pro-inflamatory cytokine interferon-gamma (IFN-γ) is central to the immunosuppressive function of hMSCs [34,35,36]. hBM-MSCs harvested from the bioreactors were cultured for 24 ± 4 h, then washed and stimulated with 10 ng/mL IFN-γ for 24 h. The cell supernatant was collected, and the kynurenine concentration was measured using a spectrophotometric assay and normalized to number of cells and days of incubation to obtain the amount of IDO secreted (expressed as pg kynurenine per cell per day). This kynurenine level was then compared to the levels from the 2D flask controls.

#### 2.5.5. Angiogenic Cytokine Secretion

MSCs have been shown to secrete cytokines which help promote tissue angiogenesis [37,38,39], therefore we quantify the secretion of various angiogenic cytokine as a measure of cell functionality. hBM-MSCs harvested from the bioreactors were cultured for 24 ± 4 h, then washed and switched to medium supplemented with 2% FBS. After 24-h incubation, the supernatant was collected and assayed for FGF, HGF, IL-8, TIMP-1, TIMP-2 and VEGF concentration using a MultiPlex ELISA (Quansys Biosciences, Logan, UT, USA). Cytokine concentration was normalized to number of cells and days of incubation to obtain cytokine secretion rates, and compared to the concentrations from the 2D flask controls.

### 2.6. Statistical Analysis

We previously quantified bioreactor-to-bioreactor variability within an experiment, and observed the following coefficient of variation values: CV < 6% for final harvest density and CV < 2% for post-bioreactor cell expansion (Figure S2). Due to these small variabilities, a ≥ 10% difference among groups of different experimental parameters was deemed biologically meaningful. A sample size of *n* = 1 bioreactor per group in these kinetic experiments therefore still allowed for the determination of the effects of the tested experimental parameters. This approach allowed for the breadth of experiments presented here. 

## 3. Results and Discussion

### 3.1. Cell and Microcarrier Inoculation Concentration in a Fixed Media Volume

**Figure 2:**
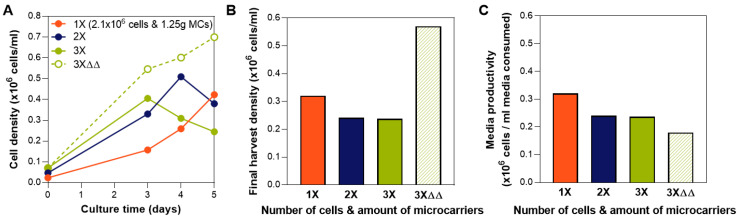
Determination of cell and microcarrier inoculation number. (**A**) Growth kinetics in bioreactors for various cell numbers and microcarrier during seeding, as well as media exchanges. Cell counts were obtained from sampling. (**B**) Final harvest density obtained from in-vessel harvest. At the highest tested cell-to-media ratio, cell growth can only be supported through media exchanges. (**C**) Media productivity comparison shows the disadvantage of media exchanges despite the high cell yield. Tested groups are 1×: 2.1 × 10^6^ cells and 1.25 g; 2×: 4.2 × 10^6^ cells and 2.5 g; 3×: 6.3 × 10^6^ cells and 3.75 g; and 3×ΔΔ: 6.3 × 10^6^ cells and 3.75 g with media exchanges on Days 3 and 4.

To first determine the maximum expansion capability in a fixed media volume in the bioreactor, we performed a fed-batch culture in bioreactors where a range of cell numbers and microcarriers amount were seeded, from 1.25 g and 2.1 × 10^6^ cells (1×) to three times the microcarriers and cell number at 3.75 g and 6.3 × 10^6^ cells (3×). At 1.25 g, the microcarriers provide 450 cm^2^ surface area in 90 mL medium, which is the recommended surface area to media volume ratio by the manufacturer. The cell density for the 1× condition is 23,333 cells/mL (4667 cells/cm^2^). The effect of media exchanges on the group with the highest cell number and microcarriers (at a concentration that far exceed the manufacturer’s recommendations) was also investigated (denoted as 3×ΔΔ in Figure 2). Among the three fed-batch conditions, we observed that bioreactor seeding with 2.1 × 10^6^ cells with 1.25 g microcarriers resulted in the highest cell yield (Figure 2A,B). In the groups where 4.2 × 10^6^ cells or 6.3 × 10^6^ cells were seeded, cell growth was limited by the media, as shown by the decrease in cell density on day 3 or day 4 (Figure 2A). With two complete media exchanges on day 3 and day 4 (instead of the bioreactor feed addition), growth of 6.3 × 10^6^ cells can be supported (Figure 2A, dotted line), justifying the media limitation in our fed-batch system at this high cell-to-media ratio. Media exchanges, however, are costly [30], resulting in low media productivity (Figure 2C), and completely untenable at larger bioreactor scales. Thus, a fed-batch paradigm is critical due to its high media productivity (Figure 1B). We therefore selected 2.1 × 10^6^ cells with 1.25 g microcarriers for seeding, which then remained consistent during evaluation of subsequent process parameters.

### 3.2. Bioreactor Seeding Strategies: Cell and Microcarrier Concentrations at Inoculation

**Figure 3:**
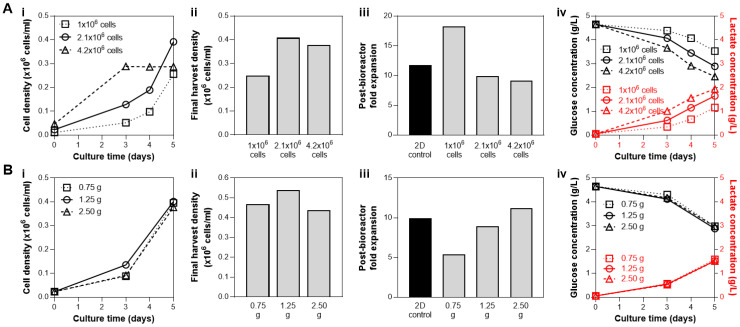
Optimization of bioreactor seeding parameters at fixed microcarrier amount (A; 1.25 g), or fixed cell number (B; 2.1 × 10^6^ cells). (**A**) Effect of cell seeding numbers on bioreactor culture yield with 1.25 g microcarriers, and (**B**) effect of microcarrier density on bioreactor culture yield with 2.1 × 10^6^ cells seeded. For both optimizations, we presented hMSC growth kinetics in bioreactors based on cell density from daily sampling (i), final density obtained from an in-vessel harvest (ii), expansion of cells harvested from the bioreactors compared to the 2D control (iii), and analysis of nutrient and waste in medium during culture (iv). Both seeding parameter studies support bioreactor seeding with 1.25 g of microcarriers and 2.1 × 10^6^ cells (4667 cells/cm^2^) to optimize for the cell yield and post-bioreactor expansion, while minimizing costs associated with materials.

The effects of cell seeding parameters, i.e., cell numbers and microcarrier surface area, were each investigated independently. First, various cell numbers were seeded into the bioreactor with 1.25 g microcarriers: 1 × 10^6^ cells (2222 cells/cm^2^ seeding density), 2.1 × 10^6^ (4667 cells/cm^2^ seeding density), and 4.2 × 10^6^ cells (9333 cells/cm^2^ seeding density). Seeding of 2.1 × 10^6^ and 4.2 × 10^6^ cells resulted in similar yield of 4 × 10^5^ cells/mL, while bioreactor seeding with 1 × 10^6^ cells resulted in the lowest yield (Figure 3(Ai,Aii)). However, a high cell number at inoculation (4.2 × 10^6^ cells) reached a growth plateau after 3 days despite the addition of a bioreactor feed on Day 3 (Figure 3(Ai)). Note that it is best practice to harvest cells during the exponential growth phase while proliferation still occurs (not during the plateau) to help ensure the maintenance of the hMSC phenotype. Inoculation with 2.1 × 10^6^ cells resulted in both the highest observed cell yield and maintained post-bioreactor cell health, as shown by the expansion of the cells harvested from the bioreactor, which is similar to that of the 2D control (Figure 3(Aiii)). Higher post-bioreactor expansion potential of the cells from the 1M group is likely due to the observed lower proliferation within the bioreactor, resulting in cells with a lower population double level (PDL). Medium analysis from all three bioreactor conditions revealed observable glucose consumption and lactate production over the culture duration (Figure 3(Aiv)), with the 4.2 × 10^6^ group with the highest nutrient consumption and waste production. Taken together, these results indicated the use of 2.1 × 10^6^ cells (23,333 cells/mL) as the cell inoculation density in subsequent studies, as after 5 days of culture the cell yield is maximized, and cell health is maintained.

With the cell to media ratio established at 2.1 × 10^6^ cells and 90 mL, the effect of seeding density was then investigated. The amount of microcarriers were varied, providing a range of available surface area for cell growth: 0.75 g (270 cm^2^), 1.25 g (450 cm^2^), and 2.5 g (900 cm^2^), which corresponds to seeding density of 7777 cells/cm^2^, 4667 cells/cm^2^, and 2333 cells/cm^2^, respectively. The growth kinetics and final cell yield after a 5-day culture were similar among the three conditions (Figure 3(Bi,Bii)). Analysis of nutrient and waste concentration in the medium also showed comparable glucose consumption/lactate production profiles in all three bioreactor conditions (Figure 3(Biv)). However, the cells harvested from the bioreactor with 0.75 g microcarriers (7777 cells/cm^2^) showed markedly reduced expansion compared to the other conditions with 1.25 g (4667 cells/cm^2^) and 2.5 g microcarriers (2333 cells/cm^2^), as well as the 2D control (Figure 3(Biii)). Thus, although bioreactor inoculation with 2.5 g microcarriers resulted in similar biological metrics (final harvest density, post-bioreactor cell expansion, and metabolite profile), 1.25 g was preferred due to the reduction in materials cost, which has notable impact when processes are scaled up to the pilot and production level (potentially hundreds to thousands of liters). 

These results indicate the use of 2.1 × 10^6^ cells and 1.25 g microcarriers for bioreactor inoculation. The formation of hMSC-microcarrier aggregates associated with these bioreactor seeding studies is shown in Figure S3, where aggregate size at Day 5 was seen to be independent of cell numbers or microcarrier amount at seeding. This qualitative assessment of the bioreactor culture looks largely similar for all of the experiments; therefore, typical pictures are not shown for the remainder of the study.

### 3.3. Bioreactor Culture Parameters: Agitation Speed, Day of Bioreactor Feed, Microcarrier Addition

We have shown that the addition of a bioreactor feed is necessary to obtain optimal cell growth (Figure 1A) without costly media exchanges (Figure 2A). With the fed-batch process in place, further optimization on the bioreactor culture process was performed. The increase of agitation speed on Day 3 was initiated due to the rapid cell growth following the feed addition, which often results in a high degree of cell aggregation (Figure 1C, Figure S3). Cell/microcarrier aggregation can be caused by cell-cell connections aided by the production of extracellular matrix (ECM) [40], which acts as an adhesive to bridge microcarriers upon collision during culture. This phenomenon started to be evident on Day 3 of the culture (Figure 1C). Subsequent increase in aggregate size may be due to increased cell number following the bioreactor feed addition and hence increased production of ECM.

Extensive cell aggregation has been mentioned to possibly correlate with the end of the exponential growth phase, as the cells within these aggregates were unable to receive sufficient nutrients and oxygen [41]. In fact, cell viability in hMSC aggregates has been shown to decrease as aggregate size increases due to limited nutrient transfer to the cells [40,42], therefore for optimal cell yield inside the bioreactor, aggregate size needs to remain small enough for it to continue being viable during culture.

**Figure 4:**
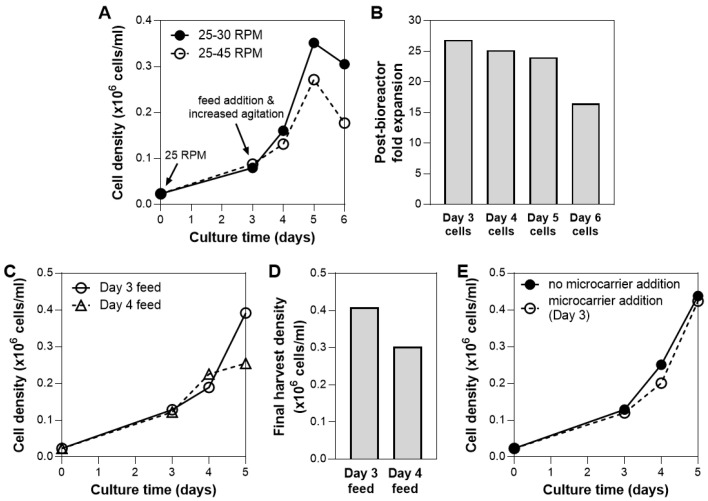
Optimization of bioreactor culture process. (**A**) Effect of bioreactor agitation speed on hMSC growth. Higher agitation speed (45 rpm) results in lower final cell yield. (**B**) Cell health following bioreactor culture. Cells harvested from the bioreactor on Day 6 showed 30% reduced expansion potential. (**C**,**D**) Effect of bioreactor feed timing on hMSC growth. Addition of feed on Day 3 results in the highest cell yield. (**E**) Effect of microcarrier addition during culture. Addition of 1.25 g of microcarriers (additional 450 cm^2^ of surface area) to increase growth surface area during culture did not influence overall hMSC growth.

To prevent cells from forming large aggregates, agitation speed can be varied. The cell/microcarrier suspension was subjected to two agitation speeds following addition of the bioreactor feed on Day 3: 30 rpm or 45 rpm. The bioreactor culture that is subjected to higher agitation speed showed slower growth after Day 3 (Figure 4A). A similar trend has been observed in other suspension bioreactor systems, where agitation above the optimal speed that keeps the cells in suspension results in a decrease in cell yield [43]. The reduction of cell growth at a higher agitation speed could be attributed to local shear-induced mechanical damage/cell detachment [8,9] or high levels of lactate production that inhibits cell growth [43].

The cell growth kinetics also showed a decline in cell density after 5 days of culture (Figure 4A). In addition, when cells sampled from each day of bioreactor culture were plated, we observed >30% decline in expansion potential for cells harvested from Day 6 compared to Day 5 (Figure 4B). To both maximize cell yield and maintain post-harvest cell health, bioreactor harvest was therefore performed on Day 5 moving forward.

Another process parameter that is critical during a fed-batch bioreactor culture is the timing of the feed addition. Investigation of the effect of dynamic nutrient feeding in bioreactors has been performed in other cell types, where feedback control on nutrient demand was utilized to optimize for efficient cell metabolism [44,45]. Similarly, in our system, the effect of feeding time was evaluated. We observed that culture with feed on Day 4 resulted in a yield that was >25% lower than culture fed on Day 3 (Figure 4C,D). As seen in the shape of the growth kinetic curves, feed addition on Day 3 is necessary to maintain exponential cell growth over 5 days, while Day 4 addition was after media limitations had led to irreversible adverse effects on growth rates. In separate experiments, we also observed that addition of feed on Day 2 or the addition of two feeds (on Day 3 and 4, or Day 2 and 4), did not result in higher cell yield compared to feed addition on Day 3 (data not shown). This was consistent with our cytokine analysis data from the bioreactor spent media, which showed rapid depletion of FGF in bioreactor culture within days (Figure S4). The bioreactor feed was therefore added on Day 3 to replenish the media in subsequent studies.

Bead-to-bead cell transfer has been demonstrated in cultures of various cell types in various bioreactor systems [46,47,48,49]. The addition of fresh, empty microcarriers to an ongoing suspension culture could therefore potentially increase yield by providing additional area for cell growth (assuming media is not the limitation). For hMSCs, it has been reported that the bead-to-bead transfer process depends on the microcarrier substrate and culture medium [48]. In this system, the addition of 1.25 g of microcarriers (additional 450 cm^2^ of surface area) on Day 3 of bioreactor culture showed no effect on cell growth (Figure 4E), possibly due to minimal cell migration between microcarriers. Complete investigation of bioprocess parameters that may allow the addition of microcarriers to in fact increase cell yield are beyond the scope of these studies.

### 3.4. Addition of Surfactant 

**Figure 5:**
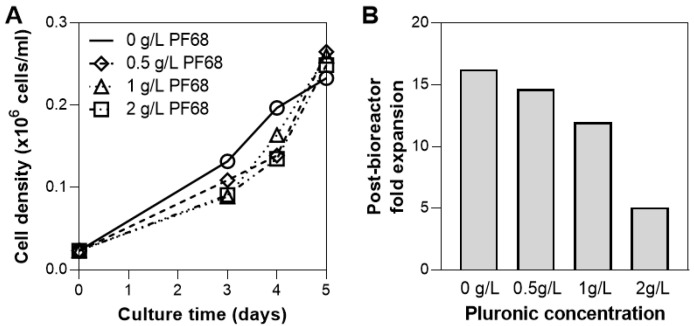
Effect of surfactants on hMSC growth in bioreactor. Addition of pluronic PF68 shows no effect on cell growth (**A**), however a high concentration of PF68 (2 g/L) markedly reduces post-bioreactor expansion (**B**).

While oxygen supply for hMSC culture at this scale (0.1 L) is sufficient through headspace gassing, sparging is often necessary to increase medium oxygenation to large-scale cultures with higher densities [50]. As bioreactors are operated at increasing working volumes, the ratio of the surface area available for headspace gassing to the culture volume decreases, therefore sparging could be implemented to meet the increasing oxygen demand [51]. The presence of gas bubbles from sparging, however, are known to cause cell damage and thus addition of a surfactant or antifoam are often implemented to reduce this damage [52,53]. We evaluated the addition of Pluronic F68 (PF68), a common surfactant used in cell culture to protect cell membranes from shear [54,55,56], at various concentrations and quantified the effect on cell growth in the bioreactor and the post-harvest cell health. Incorporation of PF68 on Day 3 at concentrations up to 2 g/L did not hinder cell growth (Figure 5A), however the use of 2 g/L of PF68 in the bioreactor culture reduced the cell expansion following bioreactor harvest by >50% compared to when 1 g/L PF68 was used (Figure 5B). Therefore, when sparging is necessary, a dosimetry study of pluronic concentration is necessary to determine the impact on post-expansion cell health, and in the case of PF68 a concentration of ≤1 g/L is recommended.

### 3.5. Bioreactor Harvest Process: Agitation Speed, Quench Hold Time, Quench Solution Temperature

**Figure 6:**
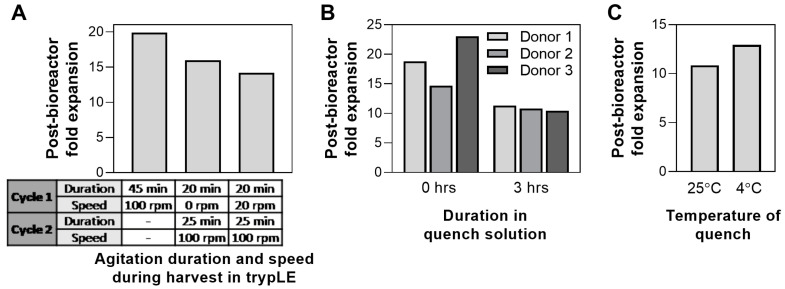
Effect of various bioreactor harvest conditions on post-harvest cell health: (**A**) agitation duration and speed during harvest in TrypLE, (**B**) duration in quench solution, and (**C**) temperature of quench solution.

Following 5-day culture, hMSCs were harvested from the bioreactor. To date, the effect of harvest parameters on hMSC health/characteristics has been mainly focused on the choice of dissociation buffer and its effect on harvest yield and cell viability [57,58,59]. Here, we evaluated additional harvest process parameters, where agitation speed and duration were varied during the cell dissociation from the microcarriers using trypLE. The effect of dissociation duration in 2D cultures has been previously shown to affect MSC phenotype [59,60]. In our system, it was observed that high agitation (100 rpm) for 45 min resulted in the highest post-harvest cell health compared to multiple cycles at various agitation speeds (Figure 6A).

Following cell detachment from the microcarriers, the enzymatic reaction was quenched by the addition of medium. Downstream processing of a large-scale bioreactor process, especially volume reduction and wash, can often take up to 4 h of processing time [61]; therefore, the effect of processing time on cell health was investigated. We compared the expansion of cells harvested from the bioreactor, where cells were plated straight after harvest (0 h) or after 3 h in quench solution. Increase of processing time to 3 h reduces the post-bioreactor cell expansion up to 54% in one donor (Figure 6B). Process development for bioreactor harvest and any further downstream processing should therefore be optimized to minimize time, particularly when scaling up to a production scale. Following harvest, the use of quench solution at 4 °C for 3 h resulted in higher post-bioreactor expansion compared to those using room temperature quench (25 °C) (Figure 6C). Although the difference is small (<20%), longer processing times likely amplify the disparity between processing at the two different temperatures. The use of a chiller is therefore recommended during downstream processing of cells from a large-scale bioreactor to optimize for cell health.

### 3.6. Validation of hMSC Expansion in Fed-batch Bioreactor Process and Maintenance of Critical Quality Attributes 

**Figure 7:**
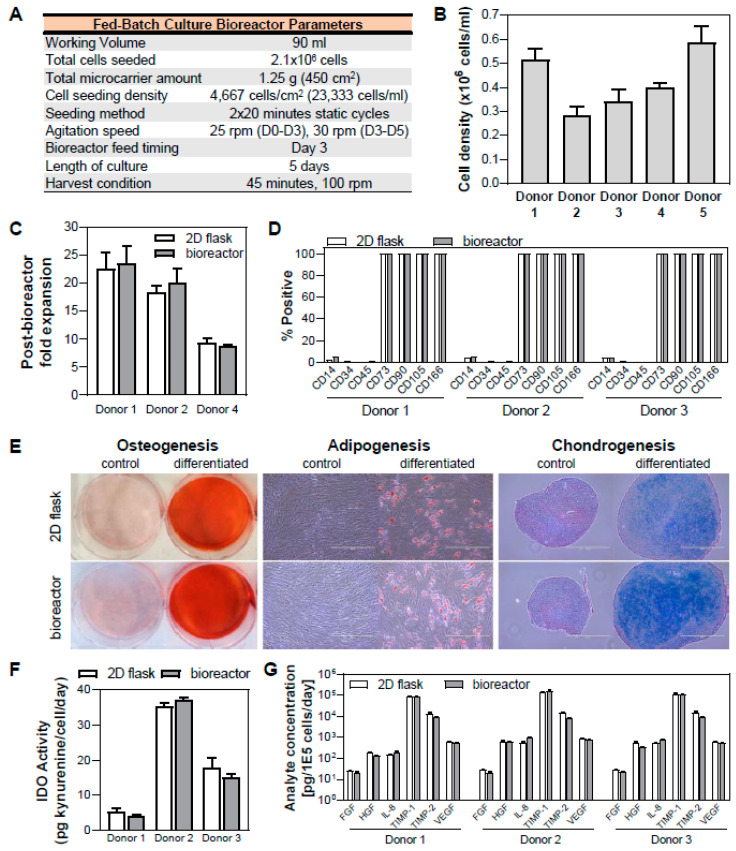
Validation of optimized fed-batch bioreactor process and hMSC characterization following bioreactor culture. (**A**) Parameters for fed-batch culture in a bioreactor. (**B**) Validation of optimized bioreactor culture process in five hMSC donors. (**C**–**G**) Verification of cell health, critical quality attributes, and functionality following bioreactor culture: XF hMSCs expanded in the bioreactor maintained their cell surface marker expression identity (**C**), tri-lineage differentiation potential to osteo-, adipo-, and chondrocytes (**E**), inducible indoleamine 2,3-dioxygenase (IDO) activity when stimulated with IFN_γ_ (**F**), and cytokine secretion profile (**G**). The health, critical quality attributes, and functionality of three hMSC donors are comparable for cells harvested from bioreactor and cells harvested from its respective 2D flask control (In panel E, Scale bar = 400 µm).

Quantification of the effects of various bioreactor process parameters on the cell yield and post-bioreactor health resulted in an optimized protocol for hMSC expansion in a vertical-wheel suspension bioreactor (Figure 7A). This fed-batch protocol was validated with expansion of 5 hMSC donors in bioreactors (Figure 7B). 

Following bioreactor culture, it is important to verify the cell health, quality, and functionality. The cell health following bioreactor culture was verified by quantifying the subsequent 2D expansion of the cells harvested from the bioreactor. For three donors, this cell expansion at P5 was comparable to each donor’s respective 2D control at similar population doubling level (PDL) (Figure 7C). In addition, the critical quality attributes (CQAs) of the cells need to be maintained and the functionality of the cells need to remain intact. Thus, we performed a general analytic panel to ensure CQAs of hMSCs, including cell identity (i.e., cell surface marker expression), trilineage differentiation potential, and functionality testing. 

We observed that cells harvested from the bioreactor maintained their CQAs of hMSC surface marker expression of CD14, CD34, CD45, CD73, CD90, CD105 and CD166 (Figure 7D) as well as osteogenic, adipogenic, and chondrogenic differentiation potential (Figure 7E), therefore meeting ISCT’s minimal criteria for defining MSCs [62]. In addition, functionality of cells from the bioreactor is also maintained, as indicated by inducible immunomodulatory potential (as measured by functional IDO activity) (Figure 7F) and angiogenic cytokine (FGF, HGF, IL-8, TIMP-1, TIMP-2, and VEGF) secretion (Figure 7G), where these values were comparable to those of cells grown in 2D culture with similar PDL. While the representative qualitative images for tri-lineage differentiation potential are shown for a single donor, images for three hMSC donors are presented in Figure S5. 

Our developed bioreactor process yielded a range of harvest densities of 2 × 10^5^–6 × 10^5^ cells/mL, which was within the reported range of hMSC yield in various microcarrier-based bioreactor systems [63]. Previous studies of hBM-MSC expansion conducted in small scale (≤1 L) microcarrier suspension bioreactors have yielded 7.5 × 10^4^–4.8 × 10^5^ cells/mL density, with most of the studies being performed under serum-containing conditions in a 7–14 day bioreactor process [63]. In xeno-free cultures, cell densities of 2 × 10^5^–3 × 10^5^ cells/mL were achieved after 14 days [63]. In comparison, similar yields were achieved in these studies within 5 days of culture using a fed-batch process.

With this developed fed-batch microcarrier hMSC culture process, scaleup is the necessary next step to meet the demands for commercial manufacturing. In addition to verifying and further optimizing these parameters at larger scales, there are other considerations in large-scale production of hMSCs in bioreactors, such as the availability of platforms for downstream processing [61,64]. For example, while separation of microcarriers from the cells during the harvest step was done using a 100 μm cell strainer in our study, this method is not feasible for processes ≥1 L for which single-use filter bag technology is more appropriate. Furthermore, a cell concentration step is required prior to the final formulation and fill of the cell product. Large volume manufacturing of hMSCs demands scalable technologies for this step such as continuous counterflow centrifugation systems and newer technologies such as the acoustic wave cell processing, all of which are commercially available.

## 4. Conclusions

We developed a fed-batch, microcarrier-based bioreactor process, which enhances media productivity and drives a cost-effective and less labor-intensive hMSC expansion process. In this study, we determined parameters for various stages of the bioreactor culture: cell number and microcarrier concentration at inoculation, agitation speed during expansion, feed timing, culture length, as well as agitation speed and duration during the harvest process. We found that bioreactor inoculation at 5 cm^2^ microcarriers surface area per mL of media and cell seeding density of 23,333 cells/mL resulted in an optimized yield and post-harvest cell health. The addition of a bioreactor feed on the third day of culture was critical to maintain the cell expansion by replenishing the mitogenic factors that were depleted from the medium. Our study resulted in an optimized hMSC culture protocol in a vertical-wheel suspension bioreactor. Our process is robust and works in multiple donors, and yields hMSCs with maintained expansion capability, phenotypic and functional properties that are comparable to traditional 2D flask cultures. The evaluated parameters were all investigated due to their relevance for larger scale production of hMSCs and parameter settings were selected both on biological response as well as system scalability to move towards production of hMSCs in larger bioreactors (>80 L). We intend to continue to scale up this process to development, pilot, and production scale bioreactors, which are critical steps to generating the cell numbers necessary for clinical therapies.

## Supplementary Materials

The following are available online at https://www.mdpi.com/2306-5354/7/3/73/s1, Figure S1: 24-hour cell attachment efficiency on microcarriers, Figure S2: Bioreactor-to-bioreactor variability within an experiment, Figure S3: hMSC cultures were sampled and monitored for cell growth on microcarriers during bioreactor culture, Figure S4: Depletion of FGF in bioreactor culture, Figure S5: Tri-lineage differentiation potential (to osteo-, adipo-, and chondrocytes) of 3 hMSC donors, comparing cells harvested from the bioreactor and each donor’s respective 2D flask control.
