# Supplementary material for: Bioreactor Parameters for Microcarrier-Based Human MSC Expansion under Xeno-Free Conditions in a Vertical-Wheel System
Source: Bioengineering (Basel). 2020 Jul 8;7(3):73. doi: 10.3390/bioengineering7030073 (PMC7552727; doi:10.3390/bioengineering7030073)
Supplement: Supplementary file 1 [file bioengineering-07-00073-s001.pdf]

Bioreactor Parameters for Microcarrier-Based Human MSC Cell Expansion under Xeno-Free Conditions in a Vertical-Wheel System

(Supplementary Materials)

Josephine Lembong, Robert Kirian, Joseph D. Takacs, Timothy R. Olsen, Lye Theng Lock, Jon A. Rowley, and Tabassum Ahsan *

RoosterBio, Inc., 5295 Westview Drive, Suite 275, Frederick, MD 21703, USA
* Correspondence: taby@roosterbio.com

Supplementary Materials
Figures S1–S5

Cell attachment efficiency in bioreactor

Following inoculation of the bioreactor, cell attachment efficiency on the microcarriers was quantified 24 hours after the end of the intermittent seeding protocol (2 × 20 minutes static cycles). The attachment efficiency is calculated as follows:

$$\text{Attachment efficiency} = \frac{\text{total viable cells attached } (t = 24 \text{ hours})}{\text{total viable cells seeded } (t = 0)} \times 100\%$$

A range of attachment efficiency was observed: 42–142%, with an average of 84%.

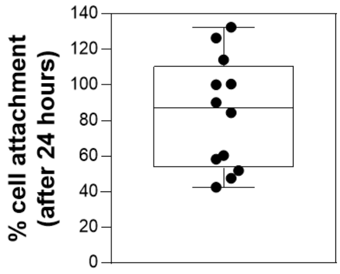

Figure S1. 24-hour cell attachment efficiency on microcarriers.

Bioreactor-to-bioreactor variability within one experiment

Three bioreactor cultures of hBM-MSCs of the same donor were run in parallel to test the variability between bioreactors within an experiment. All three bioreactors were harvested on Day 5, and the final cell density was quantified to determine bioreactor-to-bioreactor variability. The standard deviation associated with cell density among the three bioreactors were observed to be negligible (CV < 6%), justifying the use of n = 1 bioreactor per experimental group in our studies. A similar observation was made for the post-harvest P5 expansion, where the three triplicates from the three different bioreactors show negligible variability (CV < 2%).

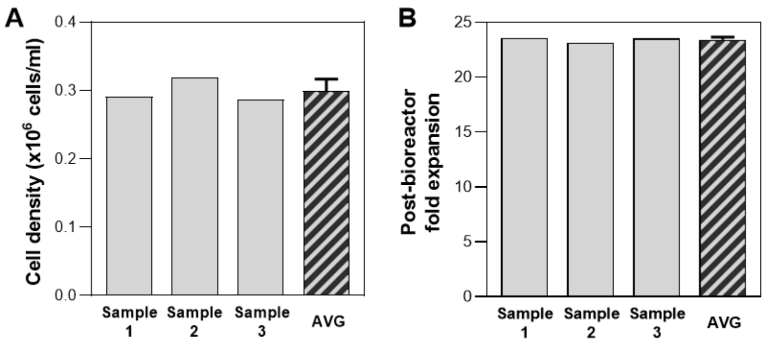

Figure S2. Bioreactor-to-bioreactor variability within an experiment. Coefficient of variation in final cell density at harvest and post-bioreactor P5 expansion are <6% of average value. Average cell density = (299,000 ± 17,500) cells/mL; average post-bioreactor fold expansion = 23.4 ± 0.25.

Formation of cell-microcarrier agglomerates during bioreactor culture

Bioreactor cultures were sampled and monitored for cell growth on microcarriers during bioreactor culture. hMSC proliferation were observed by the formation of cell-bead agglomerates during expansion. Rapid cell growth is observed in the bioreactor especially following the feed addition on Day 3, resulting in high degree of cell aggregation (Figure S3).

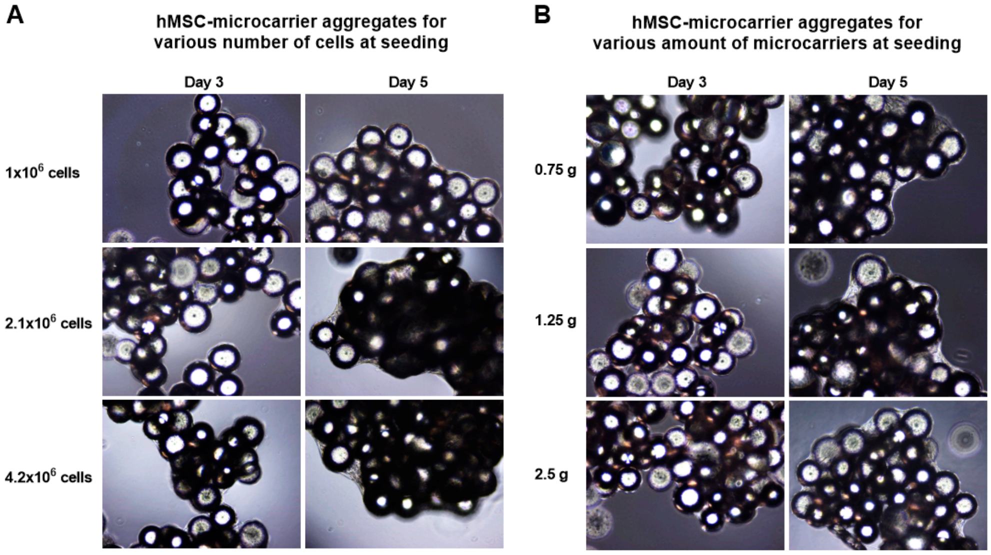

Figure S3. hMSC cultures were sampled and monitored for cell growth on microcarriers during bioreactor culture. hMSC proliferation were observed by the formation of cell-bead agglomerates during expansion.

Growth factor depletion in bioreactor culture

Analysis of cytokine concentration in spent media from bioreactor shows rapid depletion of FGF in bioreactor culture within 3 days. Bioreactor feed is therefore added on Day 3 to replenish this factor.

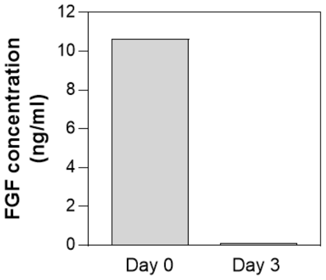

Figure S4. Depletion of FGF in bioreactor culture.

Tri-lineage differentiation of 3 hMSC donors following bioreactor culture

Three hMSC donors maintained their tri-lineage differentiation potential to osteo-, adipo-, and chondrocytes following bioreactor culture.

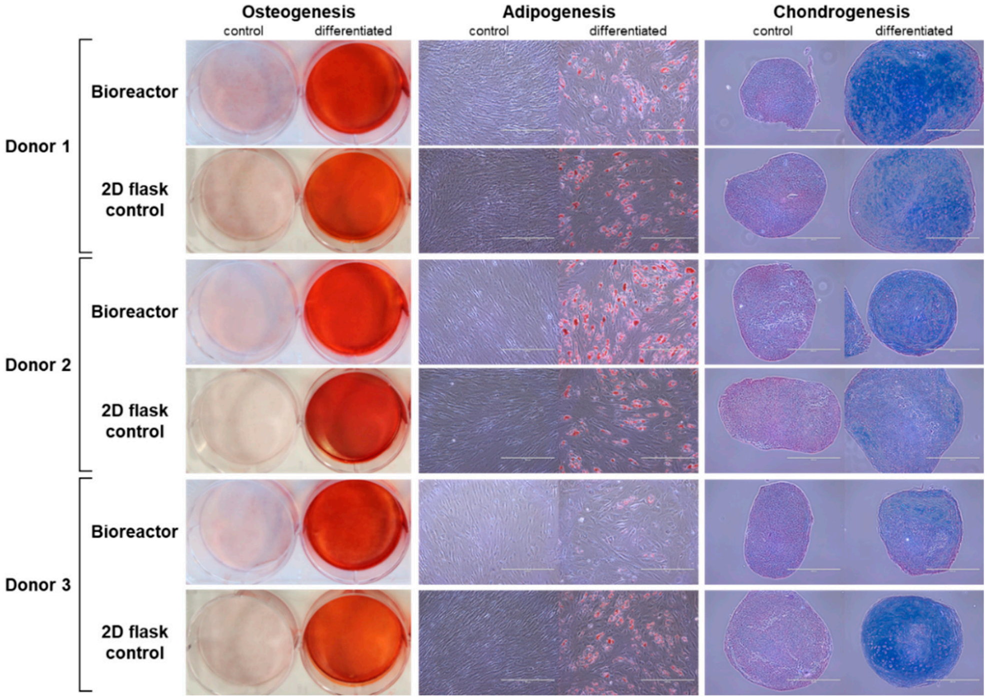

Figure S5. Tri-lineage differentiation potential (to osteo-, adipo-, and chondrocytes) of 3 hMSC donors, comparing cells harvested from the bioreactor and each donor's respective 2D flask control. All differentiated samples were positive for osteogenesis based on staining with Alizarin Red, positive for adipogenesis based on staining with Oil Red O, and positive for chondrogenesis based on staining with Alcian Blue. Scale bar = 400 μm.
